# Tick species from cattle in the Adama Region of Ethiopia and pathogens detected

**DOI:** 10.1007/s10493-021-00623-5

**Published:** 2021-04-28

**Authors:** Tafese Beyene Tufa, Silke Wölfel, Dana Zubriková, Bronislava Víchová, Martin Andersson, Ramona Rieß, Liliana Rutaihwa, André Fuchs, Hans Martin Orth, Dieter Häussinger, Torsten Feldt, Sven Poppert, Gerhard Dobler, Deon K. Bakkes, Lidia Chitimia-Dobler

**Affiliations:** 1 College of Health Science, Arsi University, Asella, Ethiopia; 2 Hirsch Institute of Tropical Medicine, Asella, Ethiopia; 3 Department of Gastroenterology, Hepatology and Infectious Diseases, Heinrich Heine University, Düsseldorf, Germany; 4 Bundeswehr Institute of Microbiology, Munich, Germany; 5 Amedes MVZ for Laboratory Medicine and Microbiology, Fürstenfeldbruck, Germany; 6 Institute of Parasitology, Slovak Academy of Sciences, Kosice, Slovakia; 7 Arlöv, Malmö, Sweden; 8 Swiss Tropical and Public Health Institute, Basel, Switzerland; 9 University Basel, Basel, Switzerland; 10 Gertrud Theiler Tick Museum, Agricultural Research Council-Onderstepoort Veterinary Research, Pretoria, South Africa; 11 Evolutionary Genomics Group, Department of Botany and Zoology, Stellenbosch University, Stellenbosch, South Africa

**Keywords:** Ticks, Tick-borne pathogens, Cattle, Ethiopia

## Abstract

Ticks will diminish productivity among farm animals and transmit zoonotic diseases. We conducted a study to identify tick species infesting slaughter bulls from Adama City and to screen them for tick-borne pathogens. In 2016, 291 ticks were collected from 37 bulls in Adama, which were ready for slaughter. Ticks were identified morphologically. Total genomic DNA was extracted from ticks and used to test for *Rickettsia* spp. with real-time PCR. Species identification was done by phylogenetic analysis using sequencing that targeted the 23S-5S intergenic spacer region and *ompA* genes. Four tick species from two genera, *Amblyomma* and *Rhipicephalus*, were identified. *Amblyomma cohaerens* was the dominant species (n = 241, 82.8%), followed by *Amblyomma variegatum* (n = 22, 7.5%), *Rhipicephalus pulchellus* (n = 19, 6.5%), and *Rhipicephalus decoloratus* (n = 9, 3.0%). Among all ticks, 32 (11%) were positive for *Rickettsia* spp. and 15 (5.2%) of these were identified as *R. africae* comprising at least two genetic clades, occurring in *A. variegatum* (n = 10) and *A. cohaerens* (n = 5). The remainder of *Rickettsia*-positive samples could not be amplified due to low DNA yield. Furthermore, another 15 (5.2%) samples carried other pathogenic bacteria: *Ehrlichia ruminantium* (n = 9; 3.1%) in *A. cohaerens*, *Ehrlichia* sp. (n = 3; 1%) in *Rh. pulchellus* and *A. cohaerens*, *Anaplasma* sp. (n = 1; 0.5%) in *A. cohaerens*, and *Neoehrlichia mikurensis* (n = 2; 0.7%) in *A. cohaerens.* All ticks were negative for *Bartonella* spp., *Babesia* spp., *Theileria* spp., and *Hepatozoon* spp. We reported for the first time *E. ruminatium*, *N. mikurensis*, *Ehrlichia* sp., and *Anaplasma* sp. in *A. cohaerens.* Medically and veterinarily important pathogens were mostly detected from *A. variegatum* and *A. cohaerens.* These data are relevant for a One-health approach for monitoring and prevention of tick-borne disease transmission.

## Introduction

Ticks are regarded as the second most important ectoparasites next to mosquitos, and act as vectors for pathogens of veterinary and medical importance (de la Fuente et al. 2008). They spread a wide-range of diseases caused by viruses, parasites, and bacteria (Mansfield et al. 2017), such as Crimean-Congo Hemorrhagic Fever (CCHF), ehrlichiosis, anaplasmosis, borreliosis, hepatozoonosis, babesiosis, and theileriosis (Mansfield et al. 2017; de la Fuente et al. 2017). This leads to ticks and tick-borne diseases (TBDs) incurring heavy economic burdens by reducing animal production and increasing treatment costs. The estimate of global financial losses due to ticks and TBDs is US $14–19 billion annually (Jabbar et al. 2015). In Tanzania, which has similar socio-economic conditions as Ethiopia, annual losses are estimated around US $364 million (Kivaria 2006).

Ticks are important vectors of *Rickettsia* spp. from the Spotted Fever Group (SFG) (Parola et al. 2013). *Rickettsia africae*, which causes African tick-bite fever (ATBF) in humans, are widespread in sub-Saharan Africa and parts of the Caribbean, and are maintained by tick populations in these regions. *Amblyomma variegatum* is the main reservoir of *R. africae*, and is abundant and widespread in sub-Saharan Africa (Maina et al. 2014). Rickettsioses and other TBDs pose underestimated risks to local inhabitants and travelers (Parola et al. 2013). Furthermore, TBDs might be underdiagnosed both in animals and humans due to limited molecular or species-specific rapid tests, especially in tropical countries like Ethiopia.

In Ethiopia, limited data are available regarding tick biodiversity (Asmare et al. 2017), despite evidence of *Rickettsia* spp. occurring in ticks (Philip et al. 1966; Parola et al. 2013) and causing disease in humans (Ramos et al. 2019). There is a lack of data regarding prevalence and significance of tick-borne infections in Ethiopia, and in the Oromia region (Kumsa et al. 2015). Surveillance to link tick species with pathogens, in combination with their respective geographic distributions as well as population dynamics and gene flow patterns, is an important process for monitoring and determining risks associated with ticks and TBDs.

Adama is located in the east Shewa Zone, Oromia Regional State, Central Ethiopia, at an altitude of 1712 m in the Great Rift Valley. As reported by city administration, up to 250 cattle per day are slaughtered in Adama for local meat consumption. Breeds of cattle include Borana and Arsi or Bale, which are indigenous breeds (Edea et al. 2013). Cattle of various ages and weights are raised in pastoral or agro-pastoral areas and then provided to merchants or butchers in Adama, either directly or after fattening for a few months.

The aim of this study was to identify tick species from bulls slaughtered in Adama City, and to investigate which pathogens they carry to assess the potential importance of these pathogens for veterinary medicine and public health.

## Material and methods

**Fig. 1:**
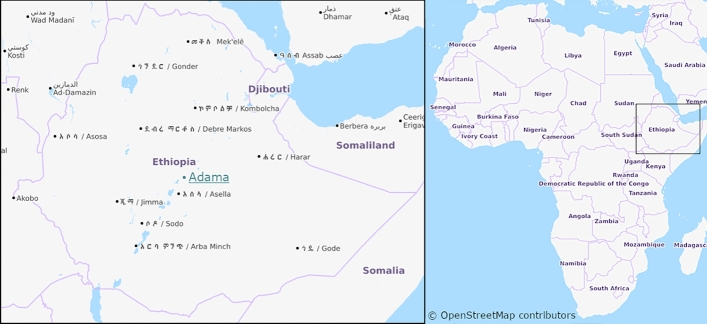
Map of Ethiopia, with a mark on Adama City. Adama is one of the largest cities found in the central part of Ethiopia

Ticks were collected from bulls ready for slaughtering at Adama City slaughterhouse in 2016 (Fig. 1). The mean age of the bulls was 6.5 years (range 5–8 years). Almost all the bulls had average body condition and they were locally bred. All ticks found on each bull ready for slaughtering were collected, and put in 20-ml Cellstar sterile tubes with pieces of grass. Ticks were later stored in 70% alcohol in Ethiopia and transported at the Hirsch Institute of Tropical Medicine (HITM) Laboratory, before being shipped for investigation of pathogens to the Bundeswehr Institute of Microbiology in Munich, Germany. Ticks were exported to Germany with ethical approval from the Ethiopian Biodiversity Institute by the reference letter EBI 71/2082/2014. Ticks were identified to the species level using morphological characters according to Voltzit and Keirans (2003) and Walker et al. (2000, 2003).

Total nucleic acid was extracted from individual ticks with the MagNA Pure LC RNA/DNA Kit (Roche, Mannheim, Germany) in a MagNA Pure LC instrument (Roche). Ticks were tested for *Rickettsia* spp. with a real-time PCR known to amplify part of the *gltA* gene of various *Rickettsia* spp. (Wölfel et al. 2008). Thereafter, for further molecular characterization and to identify *Rickettsia* species, conventional PCRs were conducted targeting the 23S-5S intergenic spacer region PCR (Chitimia-Dobler et al. 2018) as well as an *ompA* PCR (Fournier et al. 1998). Amplification of *Bartonella* spp. was achieved with a conventional PCR targeting a part of the *ssrA*-gene as published previously (Diaz et al. 2012). Detection of *Anaplasma* spp., *Ehrlichia* spp., and *Neoehrlichia* spp. was performed with primers that amplify a part of the 16S rRNA gene (Krücken et al. 2013). Furthermore, ticks were examined for the presence of the tick-borne apicomplexan parasites *Babesia* spp., *Theileria* spp., and *Hepatozoon* spp. using an earlier published PCR-assay that amplifies a part of the 18S rRNA gene (Andersson et al. 2017).

Subsequent Sanger sequencing of amplicons was conducted by an exterior supplier (GATC Biotech, Konstanz, Germany). Sequences were analyzed using BioEdit Alignment Editor v.7.1.1 (Hall 1999) and compared with sequences deposited in the GenBank database of the National Centre for Biotechnology Information (NCBI) using the Basic Local Alignment Search Tool (BLAST; Altschul et al. 1990). To identify *Rickettsia* species, phylogenetic analyses of the 23S-5S intergenic spacer and *ompA* genes were performed. Nucleotide sequence data were aligned using MAFFT (Q-INS-i, 200PAM/k = 2, Gap opening penalty 1.53) (Katoh et al. 2002), with final alignments comprising 238 bp (23S-5S) and 682 bp (*ompA*). Alignment was inspected to ensure sequences were in reading frame. Optimal nucleotide substitution models were selected using BIC calculations in W-IQ-TREE (Trifinopoulos et al. 2016), and were determined as TN92 for both alignments. Maximum likelihood analysis was performed in MEGA v.7.0.14 (Kumar et al. 2016) with 1000 bootstraps.

## Results

In total, 291 ticks from 37 bulls were collected, and 4–28 ticks were collected per bull. Most ticks collected were adults, with 22 females, 262 males, and seven nymphs. Ticks were identified as *Amblyomma cohaerens* (n = 241, 82.8%), *A. variegatum* (n = 22, 7.5%), *Rhipicephalus pulchellus* (n = 19, 6.5%) and *Rhipicephalus decoloratus* (n = 9, 3.0%). *Amblyomma cohaerens* was represented by 6 nymphs, 11 females, and 224 males, whereas *A. variegatum* was represented by only 1 nymph, 2 females, and 19 males. Of the two *Rhipicephalus* species only adults were collected, comprising *Rh. decoloratus* females and *Rh. pulchellus* males. All ticks collected were partly engorged.

**Fig. 2:**
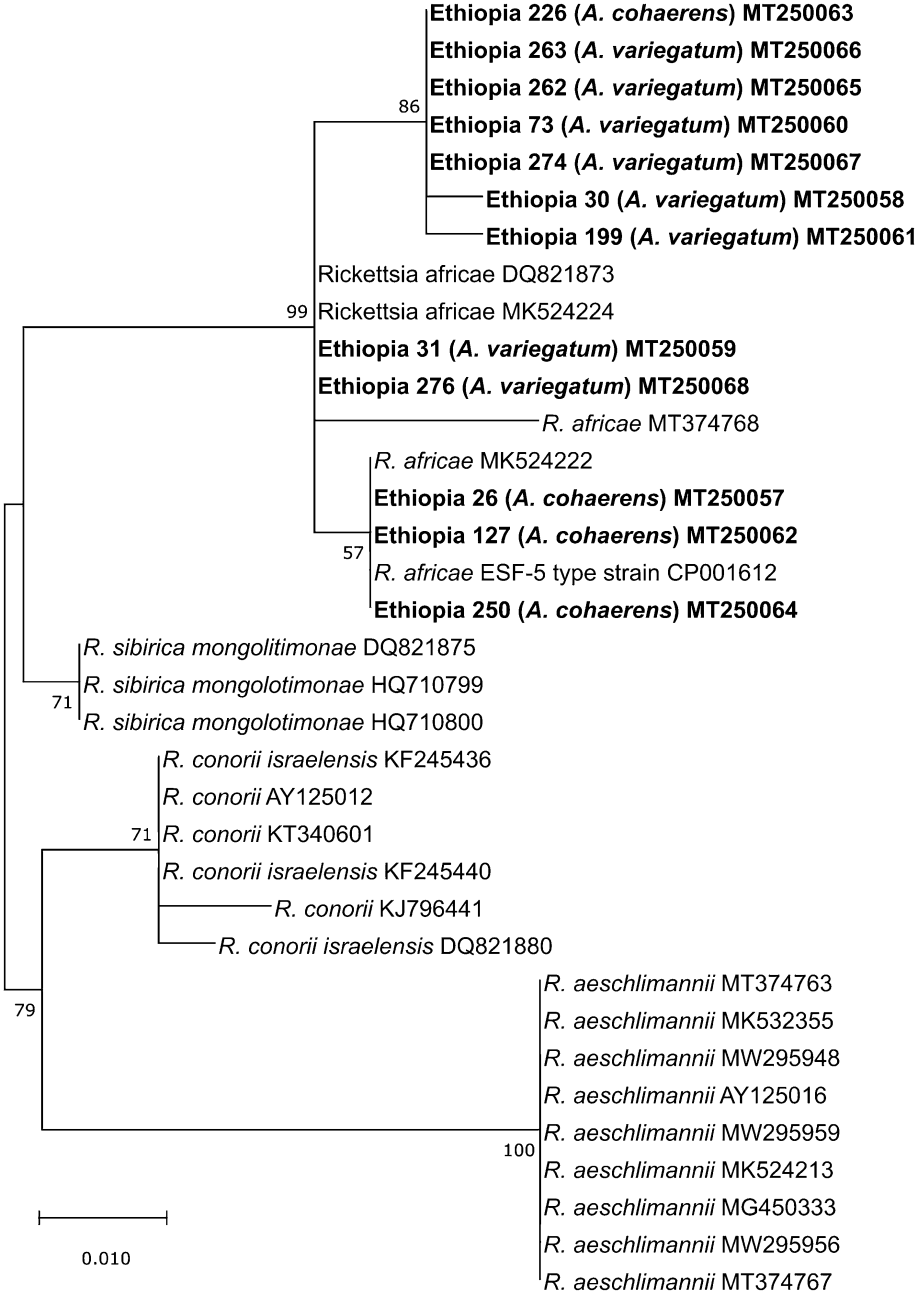
Maximum likelihood phylogenetic analysis of 23S-5S intergenic spacer sequences for *Rickettsia.* Bolded terminals refer to sequences generated in this study. Sample ID, host and GenBank accession numbers are indicated. Nodal values indicate bootstrap support using 1000 replicates

**Fig. 3:**
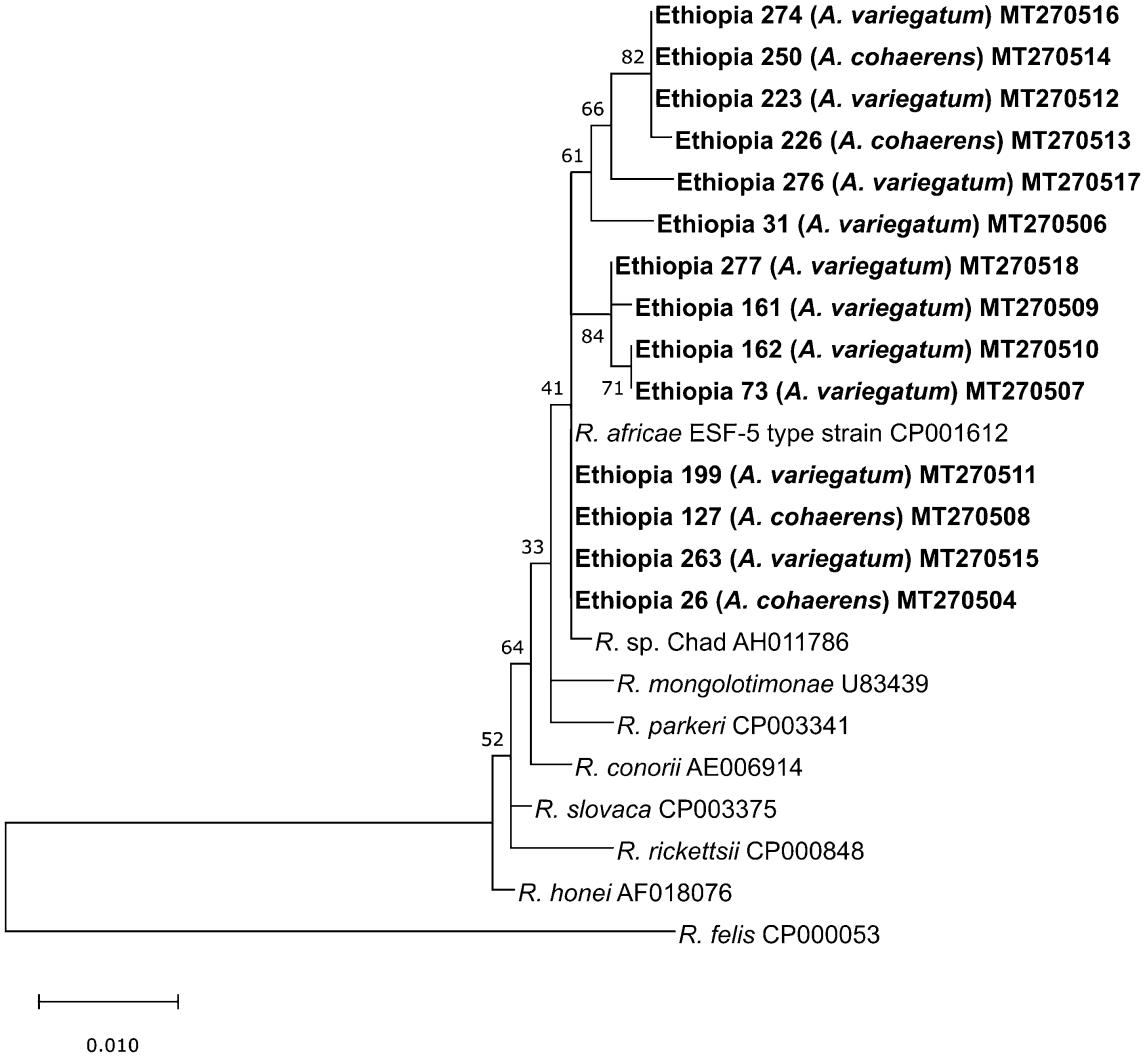
Maximum likelihood phylogenetic analysis of *ompA* sequences for *Rickettsia*. Bolded terminals refer to sequences generated in this study. Sample ID, host and GenBank accession numbers are indicated. Nodal values indicate bootstrap support using 1000 replicates

From the 291 tested ticks, 32 (11%) were positive in pan-*Rickettsia* (pan-Rick) real-time PCR. Further investigations were possible for only 15 (5.2%) samples, because the remaining 17 samples yielded too little DNA for amplification. These 15 samples comprised 10 *A. variegatum* and five *A. cohaerens.* Only 12 samples could be amplified in the 23S-5S intergenic spacer region PCR (~ 350 bp), whereas 15 samples could be amplified in the *ompA* gene (~ 800 bp). Both amplicon targets investigated matched most closely with *R. africae* in all samples. Sequence identity to the type strain *R. africae* ESF-5 (CP001612) ranged 99.19–100% for *ompA* (accession numbers: MT270504–MT270518), and 98.57–100% for the 23S-5S intergenic spacer region (accession numbers MT250057–MT250068). Phylogenetic analysis revealed circulation of at least two distinct genetic clades of *R. africae* in the Oromia region, with good bootstrap support > 70% among 5S-23S sequences (Fig. 2). Furthermore, these two clades were present in both *A. cohaerens* and *A. variegatum*. However, these clades were less distinct among *ompA* sequences (Fig. 3).

Furthermore, 21/291 (7.2%) samples could be amplified in the 16S rRNA gene (~ 250 bp) could be amplified. Sequence analysis revealed nine *Ehrlichia ruminantium* all detected in *A. cohaerens*, along with three *Ehrlichia* sp. detected in *Rh. pulchellus* (n = 2) and *A. cohaerens* (n = 1). One *Anaplasma* sp. was detected in *A. cohaerens*, and two *Neoehrlichia mikurensis* were detected in *A. cohaerens*. Six samples did not provide results due to low DNA content. All ticks were negative for *Bartonella* spp., *Babesia* spp., *Theileria* spp., and *Hepatozoon* spp. Details on tick-borne pathogens detected in each tick species are given in Table 1.

**Table 1:** Tick species and stages which tested positive for various tick-borne pathogens

Nos.	Tick species	Tick stage			*Rickettsia* spp.^+^	*Anaplasma* sp.	*Ehrlichia* sp.	*Ehrlichia ruminantium*	*Neoehrlichia mikurensis*
		Male	Female	Nymph					
1	*Amblyomma cohaerens*	+					+		
2	A. cohaerens*^#^	+			+				
3	A. variegatum*	+			+				
4	A. variegatum*^#^	+			+				
5	*A. cohaerens*	+			+			+	
6	*A. cohaerens*	+			+			+	
7	A. variegatum*^#^	+			+				
8	*A. cohaerens*	+						+	
9	*A. cohaerens*	+						+	
10	*A. cohaerens*	+						+	
11	*A. cohaerens*	+						+	
12	A. cohaerens*^#^	+			+				
13	*Rhipicephalus pulchellus*	+					+		
14	*A. cohaerens*		+						+
15	*A. variegatum*	+			+				
16	*A. variegatum*^#^	+			+				
17	*A. variegatum*^#^		+		+				
18	*A. cohaerens*	+				+			
19	*A. cohaerens*	+						+	
20	*A. variegatum*	+			+				
21	*A. variegatum*	+			+				
22	A. variegatum*^#^	+			+				
23	*A. cohaerens*			+	+				
24	*A. cohaerens*	+							+
25	*A. cohaerens*	+						+	
26	*A. variegatum*		+		+				
27	*A. variegatum*^#^	+			+				
28	*A. cohaerens*	+			+				
29	A. cohaerens*^#^	+			+				
30	*A. cohaerens*	+						+	
31	*Rh. pulchellus*	+					+		
32	*A. cohaerens*	+			+				
33	*A. cohaerens*	+			+				
34	A. cohaerens*^#^	+			+				
35	*Rh. pulchellus*	+			+				
36	A. variegatum*	+			+				
37	A. variegatum*	+			+				
38	A. variegatum*^#^	+			+				
39	*A. variegatum*	+			+				
40	A. variegatum*^#^	+			+				
41	*A. variegatum*	+			+				

^+^*Rickettsia* spp. represent all the positive specimens in screening pan-Rick PCR. Not all positive specimens in pan-Rick PCR could be further analyzed and *Rickettsia* species identified

*Positive specimens for which *Rickettsia africae* was identified using 23S-5S intergenic space gene (Fig. 2)

^#^Positive specimens for which *R. africae* was identified using *ompA* gene (Fig. 3)

## Discussion

Ticks are responsible for the transmission of many pathogens both in humans and animals (de la Fuente et al. 2017; Mansfield et al. 2017). The majority of these pathogens appear in tropical countries where environmental conditions are favorable for ticks. Unfortunately, resources for surveillance and monitoring are often inadequate in many of these tropical countries (Gubler 2009). In Ethiopia, limited information is available regarding the prevalence of zoonotic TBDs in ticks (Philip et al. 1966; Parola et al. 2013; Ramos et al. 2019).

Ticks collected from bulls in the Oromia region were investigated for the prevalence of tick-borne pathogens. Ticks belonging to two genera (*Amblyomma* and *Rhipicephalus*) and four species (*A. cohaerens*, *A. variegatum*, *Rh. pulchellus*, and *Rh. decoloratus*) were collected. *Amblyomma cohaerens* was the dominant (82.8%) tick species, followed by *A. variegatum* (7.5%), and *Rh. pulchellus* (6.5%). The geographic distribution of *A. cohaerens* is generally limited to the western parts of Ethiopia, which receives heavy rains for most of the year (Mekonnen et al. 2001). Conversely, *A. variegatum* is the most widespread member of the genus in sub-Saharan Africa. The northern border of this species lies in West Africa and extends from the Mauritania–Senegal border eastwards into most of Ethiopia. *Rhipicephalus pulchellus*, commonly known as the zebra tick, often infests a wide range of hosts, including humans. The species occur in dry areas such as savanna, steppe, and desert, and is abundant in North East Africa (Walker et al. 2003). *Rhipicephalus* (*Boophilus*) *decoloratus*, commonly known as the blue tick, is the most widespread and abundant species of the one-host cattle ticks in Africa. In East Africa and southern Africa, it occurs alongside *Rh*. (*B*.) *microplus* (Walker et al. 2003).

In this study, evidence of pathogenic bacteria (*R. africae*, *E. ruminantium*, *Ehrlichia* sp., *Anaplasma* sp., and *N. mikurensis*) was found in only three of the four collected tick species. We detected 32 (11%) *Rickettsia*-positive samples, from which 15 (5.2%) were confirmed as *R. africae* that comprise at least two distinct genetic clades. These two clades are distinct among 5S-23S sequences (Fig. 2), but less distinct among *ompA* sequences (Fig. 3) due to higher nucleotide conservation likely as a result of the gene coding for outer membrane proteins that are under selection (Nunes et al. 2009).

In Ethiopia, *Rickettsia* spp. have previously been reported in ticks (Kumsa et al. 2012, 2015). The results indicate that *A. variegatum* is the main reservoir for *R. africae*, whereas *A. cohaerens* likely play a role in maintaining the natural transmission cycle. Our findings support a report from western Kenya, in near proximity to the Ethiopian border, which indicated a high level of *R. africae* in *A. variegatum* ticks (Maina et al. 2014). Dependable identification of *Rickettsia* spp. is crucial for correct diagnosis, management, and prevention of the resulting diseases in animals as well as humans. *Amblyomma variegatum*, which is a documented vector of *R. africae* and one of the predominant tick species in western Ethiopia (Bako area) (Kumsa et al. 2014), has been shown to feed on humans (Mediannikov et al. 2010). Our finding is in line with data reported by Kemal et al. (2016) in which *A. cohaerens* was the most predominant tick species infesting cattle in the eastern part of Ethiopia. The noticeably low prevalence of *R. africae* (5/241; 2.1%) in *A. cohaerens* is also in line with previous reports that indicated low levels of SFG rickettsiae (Kumsa et al. 2012; Burgdorfer et al. 1973; Philip et al. 1966). However, the greater abundance of *A. cohaerens* compared with *A. variegatum* indicate they may provide a subtle but important link in maintaining circulation of *R. africae* in Ethiopia. Increasing the host range of *R. africae* to include *A. cohaerens*, albeit with low prevalence, can enable the pathogen to persist in regions that normally limit *A. variegatum* abundance*.*

The role of *Rhipicephalus* ticks in maintaining and transmitting *R. africae* is still not clear. However, infection rates ranging between 0.4 and 5% in *Rh. evertsi evertsi* and between 5 and 77% in *Rh. decoloratus* were previously reported in some sub-Saharan African countries (Parola et al. 2003). Likewise, *Rickettsia* spp. have also been reported in Ethiopia’s neighboring countries such as Sudan and the Central African Republic, as well as in other countries in the region (e.g., Uganda and Djibouti) with prevalence of up to 21.6% (Dupont et al. 1994; Mura et al. 2008; Shuaib et al. 2020). It is important to note that *A. variegatum* and *A. cohaerens* have been reported to feed on humans (Dantas-Torreset al. 2012; Jongejan and Uilenberg 2004; Parola and Raoult 2001), which increases the risk of zoonotic transmission of *R. africae* to humans. In tropical countries like Ethiopia, *Rickettsia* infections might be underdiagnosed both in animals and humans due to the limited accessibility of reliable and species-specific diagnostic assays.

Furthermore, 21/291 (7.2%) ticks carried other bacteria as follows: *E. ruminantium* in *A. cohaerens*, *Ehrlichia* sp. in *Rh. pulchellus* (n = 2) and *A. cohaerens* (n = 1), and *Anaplasma* sp. and *N. mikurensis* in *A. cohaerens.* Teshale et al. (2016) reported *Anaplasma ovis* and *Anaplasma* spp., *E. ruminantium* and *Ehrlichia* spp. from *Rh. decoloratus*, whereas only *A. ovis* was detected in *Rh. evertsi evertsi*; *Rickettsia* spp. and *R. africae* were found in both tick species. *Ehrlichia ruminantium*, the causative agent of heartwater, was detected in *A. variegatum* in other studies (Bekker et al. 2002; Faburay et al. 2007; Robinson et al. 2009), but not in *A. cohaerens*. Although it has been demonstrated that *A. cohaerens* can act as a vector for the heartwater agent in laboratory settings (Walker and Olwage 1987), it has so far not been detected in field-collected *A. cohaerens*. Our results demonstrate the veterinary and medical importance of *A. cohaerens* given its high abundance, as well as the high diversity of associated pathogens including *R. africae* (five positives), *E. ruminantium* (nine positives), *Ehrlichia* sp. (one positive), *Anaplasma* sp. (one positive), and *N. mikurensis* (two positives).

To our knowledge, this is the first detection of *N. mikurensis* in ticks from Ethiopia and in *A. cohaerens*. There is only one report of *N. mikurensis* in Africa, in *Rhipicephalus sanguineus* and *Haemaphysalis leachi* collected from dogs in Nigeria (Kamani et al. 2013). This finding may have important relevance for public health. *Neoehrlichia mikurensis* can cause high fever followed by thrombotic or hemorrhagic events with possible mortality if left untreated in humans (von Loewenich et al. 2010).

All ticks were negative for *Bartonella* spp., *Babesia* spp., *Theileria* spp., and *Hepatozoon* spp. Although *Rh. decoloratus* is well known to transmit *Babesia bigemina* and *Anaplasma marginale* (Walker et al. 2003), these pathogens were not detected in this study, possibly due to the low abundance of this tick species.

In conclusion, it seems that *A. cohaerens* can carry more pathogenic bacteria than was known before. *Ehrlichia ruminatium*, *Anaplasma* sp. and *N. mikurensis* were detected only in *A. cohaerens*. Some of the detected pathogens are known for their medical (*R. africae*, *N. mikurensis*) and veterinary importance (*E. ruminantium*). Altogether, our data are relevant for a One-health approach in future to prevent transmission of tick-borne diseases.
